# First-line immune checkpoint inhibitors combined with chemotherapy in advanced large-cell neuroendocrine carcinoma of the lung: a real-world retrospective study

**DOI:** 10.3389/fonc.2025.1709544

**Published:** 2025-11-18

**Authors:** Beibei Ji, Wei Luan, Rula Sha, Wenxin Li

**Affiliations:** Department of Medical Oncology, Inner Mongolia People’s Hospital, Hohhot, Inner Mongolia, China

**Keywords:** real-world study, LCNEC, immunotherapy, PD-1/PD-L1 inhibitor, survival, prognosis

## Abstract

**Background:**

Large-cell neuroendocrine carcinoma of the lung (LCNEC) shares clinicopathological features with both small cell lung cancer (SCLC) and non-small cell lung cancer (NSCLC). Owing to its neuroendocrine characteristics, the treatment of LCNEC often follows paradigms established for SCLC. Immune checkpoint inhibitors (ICIs) have become the standard first-line therapy for extensive-stage SCLC (ES-SCLC), but evidence supporting the use of ICIs in advanced LCNEC remains limited. This study aimed to evaluate the efficacy and prognosis of first-line ICIs in patients with advanced LCNEC.

**Methods:**

We retrospectively analyzed 31 patients with stage IV LCNEC treated at Inner Mongolia People’s Hospital from January 2019 to December 2024. Of these, 14 patients received ICIs plus platinum-based chemotherapy (the ICIs + Chemo group), and the other 17 patients received chemotherapy alone (the Chemo group). Progression-free survival (PFS), overall survival (OS), objective response rate (ORR), disease control rate (DCR), and treatment-related adverse events(AEs) were compared between the two groups.

**Results:**

After a median follow-up of 24 months, the ICIs + Chemo group demonstrated significantly longer median PFS (10.5 months [95% CI, 7.6-12.4] vs. 6.0 months [95% CI, 4.3-7.7]; *p=*0.035) and median OS (15.0 months [95% CI, 11.4-18.6] vs. 11.0 months [95% CI, 9.3-12.6]; *p=*0.036) compared to the Chemo group. Multivariate Cox regression showed that the ICIs + Chemo group reduced the risk of progression by 49% (HR = 0.51; 95% CI, 0.28-0.92; *p=*0.026) and death by 45% (HR = 0.55; 95% CI, 0.30-1.01; *p=*0.054). The ORR and DCR were 50.0% and 85.7% in the ICIs + Chemo group, versus 29.4% and 76.5% in the Chemo group, respectively. Immune-related adverse events (irAEs) in the ICIs + Chemo group were grade 1–2, with no grade 3 or higher adverse events observed.

**Conclusion:**

This study was based on real-world data from northern China. Preliminary findings suggest that ICIs combined with chemotherapy may be a promising treatment option for patients with advanced LCNEC, with potential survival benefits. However, as a single-center retrospective study with a limited sample size, further multi-center and large-sample prospective clinical trials are warranted to validate these results.

## 1 Introduction

Lung cancer is broadly classified as non-small cell lung cancer (NSCLC) and small cell lung cancer (SCLC). SCLC is characterized by rapid growth, high aggressiveness, a propensity for early recurrence and metastasis, and a generally poor prognosis. Approximately two-thirds of patients with SCLC are diagnosed at the extensive stage (ES-SCLC). For ES-SCLC treated primarily with chemotherapy, the median overall survival is only 8–12 months, with 1-year and 2-year survival rates of 29.4% and 7.0%, respectively, and a 5-year survival rate of less than 1% (1–3). Prior to the advent of ICIs, chemotherapy was the mainstay of treatment for SCLC. The IMpower133 trial was the first phase III study to demonstrate a significant improvement in both PFS and OS with the addition of immunotherapy to chemotherapy in ES-SCLC. Based on the findings of this study, the therapeutic paradigm for ES-SCLC has undergone substantial changes, with the standard first-line treatment shifting from traditional platinum-based chemotherapy to immunotherapy plus chemotherapy (4).

Neuroendocrine neoplasms (NENs) represent a highly heterogeneous group of tumors that arise from peptidergic neurons and neuroendocrine cells, and can occur in virtually all organs. Based on histopathological differentiation, NENs are classified into well-differentiated neuroendocrine tumors (NETs) and poorly differentiated neuroendocrine carcinomas (NECs). NETs include typical carcinoids and atypical carcinoids, whereas NECs comprise small cell neuroendocrine carcinoma (SCNEC) and large cell neuroendocrine carcinoma (LCNEC) (5). SCNEC may originate in multiple organs (e.g., pancreas, bladder, cervix, and prostate), and in the lung cancer, SCLC is itself a form of SCNEC. The distinct molecular and pathological profiles of SCNEC and LCNEC characterize them as among the most aggressive histological subtypes of lung cancer, characterized by poor prognosis and low survival rates (6). The incidence of LCNEC in the general population is approximately 0.0003% and shows an increasing trend. Among patients with LCNEC, 53.6% to 57.8% are male, and the mean age at diagnosis is around 66 years. Additionally, 52.6% to 54.6% of LCNEC cases are diagnosed at stage IV (7, 8). Commonly mutated genes in LCNEC include TP53, RB1, STK11, KEAP1, and RAS (KRAS/NRAS/HRAS), whereas alterations in driver genes such as EGFR, ALK, and MET occur at a relatively low frequency, thereby limiting the utility of targeted therapies (8–10). At present, both the National Comprehensive Cancer Network (NCCN) guidelines and the Chinese Society of Clinical Oncology (CSCO) guidelines recommend platinum-based chemotherapy as the standard first-line systemic treatment, primarily following regimens established for SCLC, with NSCLC-based regimens considered as alternatives in selected cases (11). Retrospective analyses have demonstrated that SCLC-based chemotherapy regimens are superior to NSCLC-based regimens, both in the adjuvant setting for early-stage disease and in the palliative setting for advanced disease (12).

ICIs as monotherapy have shown limited efficacy in advanced LCNEC. Accordingly, recent researches have primarily focused on combining immune checkpoints blockade targeting different pathways or integrating ICIs with other classes of agents. For patients with stage I–III LCNEC, surgery combined with chemotherapy remains the optimal treatment approach (13). But for patients with stage IV LCNEC, given its neuroendocrine characteristics, chemotherapy provides greater benefit than other therapeutic approaches (14). The treatment for stage IV LCNEC is generally aligned with SCLC regimens, most commonly etoposide plus platinum (15), with irinotecan plus platinum considered as an alternative option (16). ICIs have achieved transformative advances across multiple solid tumors, particularly in lung cancer, where they have become the standard first-line therapy for SCLC and for NSCLC lacking actionable driver mutations. However, clinical evidence regarding the use of ICIs in advanced LCNEC remains limited, and their therapeutic potential in this setting has yet to be fully elucidated. On this basis, our study aimed to preliminarily assess the potential of ICIs as first-line therapy in patients with advanced LCNEC by comparing the efficacy of ICIs plus chemotherapy with chemotherapy alone.

## 2 Patients and methods

### 2.1 Patients

We retrospectively collected clinical data of patients diagnosed with stage IV LCNEC at Inner Mongolia People’s Hospital between January 2019 and December 2024.

Inclusion criteria:

1. Confirmed LCNEC pathology reviewed by ≥2 pathologists;
2. Stage IV disease;
3. Treated with either platinum-based chemotherapy alone or in combination with ICIs (≥1 cycle);
4. At least one measurable lesion on CT with a unidimensional diameter.

Exclusion criteria:

1. Age <18 years;
2. Incomplete demographic or treatment-related data.

### 2.2 Treatment

#### 2.2.1 Immune checkpoint inhibitors: agents and administration

PD-1 agents: Sintilimab, 200 mg intravenously every 3 weeks; Tislelizumab, 200 mg intravenously every 3 weeks; Toripalimab, 3 mg/kg intravenously every 2 weeks; Seplulimab, 4.5 mg/kg intravenously every 3 weeks; Penpulimab, 200 mg intravenously every 2 weeks.

PD-L1 agents: Atezolizumab, 1200 mg intravenously every 3 weeks; Durvalumab, 1500 mg intravenously every 3 weeks; Adebrelimab, 20 mg/kg intravenously every 3 weeks.

PD-1/CTLA-4 agent: Cadonilimab, 375 mg intravenously every 2 weeks.

#### 2.2.2 Chemotherapy: agents and administration

Chemotherapy regimens were primarily platinum-based combinations, including etoposide plus platinum, irinotecan plus platinum, and albumin-bound paclitaxel plus platinum. Chemotherapy dosing was as follows:

Etoposide, 100 mg/m² intravenously on day 1 of a 3-week cycle;
Irinotecan, 65 mg/m² intravenously on day 1 of a 3-week cycle;
Albumin-bound paclitaxel, 125 mg/m² intravenously on days 1 and 8 of a 3-week cycle;
Cisplatin, 75 mg/m² intravenously on day 1 of a 3-week cycle;
Carboplatin, AUC 5–6 intravenously on day 1 of a 3-week cycle;
Lobaplatin, 30 mg/m² intravenously on day 1 of a 3-week cycle.
Drug dosages and manufacturers are detailed in **Table 1**.

**Table 1:** Treatment regimens for immunotherapy and chemotherapy.

Drug name	Dose	Frequency	Administration
PD-1 Inhibitors			
Sintilimab	200 mg	Every 3 weeks	Innovent Biologics (Suzhou) Co. Ltd.
Tislelizumab	200 mg	Every 3 weeks	Guangzhou BeOne Medicines Ltd.
Toripalimab	3 mg/kg	Every 2 weeks	Shanghai Junshi Biosciences Co., Ltd.
Seplulimab	4.5 mg/kg	Every 3 weeks	Shanghai Henlius Biopharmaceuticals Co., Ltd.
Penpulimab	200 mg	Every 2 weeks	Nanjing Shunxin Pharmaceutical Co., Ltd.
PD-L1 Inhibitors			
Atezolizumab	1200 mg	Every 3 weeks	Roche Diagnostics GmbH.
Durvalumab	1500 mg	Every 3 weeks	Catalent Indiana, LLC.
Adebrelimab	20 mg/kg	Every 3 weeks	Suzhou Suncadia Biopharmaceuticals Co., Ltd.
PD-1/CTLA-4 Inhibitors			
Cadonilimab	375 mg	Every 2 weeks	Akeso Biopharma Co., Ltd.
Chemotherapy			
Etoposide	100 mg/m²	Every 3 weeks	Jiangsu Hengrui Pharmaceuticals Co.,Ltd.
Irinotecan	65 mg/m²	Every 3 weeks	Hainan Jin Rui Pharmaceutical Co.,Ltd.
Nab-paclitaxel	125 mg/m² (d1, d8)	Every 3 weeks	Qilu Pharmaceutica Hainanco Ltd.
Cisplatin	75 mg/m²	Every 3 weeks	Qilu Pharmaceutica Hainanco Ltd.
Carboplatin	AUC = 5-6	Every 3 weeks	Qilu Pharmaceutical Co., Ltd.
Lobaplatin	30 mg/m²	Every 3 weeks	Hainan Chang’an International Pharmaceutical Co. Ltd.

Administration: All drugs are administered via intravenous infusion.

### 2.3 Data collection

Patients were divided into two groups: 14 patients with advanced LCNEC received ICIs combined with chemotherapy (the ICIs + Chemo group), and 17 patients received platinum-based combination chemotherapy (the Chemo group). Collected data included baseline demographic characteristics (age, sex, smoking status, and ECOG performance status) and the sites of metastasis at diagnosis (lung, liver, brain, bone, adrenal glands, pleura, soft tissue, spleen, etc.), as well as whether thoracic radiotherapy was administered. Pre-treatment laboratory assessments were conducted, including lactate dehydrogenase (LDH), serum sodium, neuron-specific enolase (NSE), and pro-gastrin-releasing peptide (ProGRP). Treatment regimens, adverse events, and follow-up assessments were also recorded.

Efficacy indexes included DCR and ORR, which were evaluated according to the Response Evaluation Criteria in Solid Tumors (RECIST) version 1.1. PFS was defined as the time from treatment initiation to disease progression or death from any cause, and OS was defined as the time from treatment initiation to death from any cause. The last follow-up was conducted on December 31, 2024. Diagnosis and treatment were performed in accordance with the Chinese Guidelines for the Diagnosis and Treatment of Primary Lung Cancer (2022 edition), and LCNEC classification followed the World Health Organization (WHO) Classification of Thoracic Tumors, 5th edition. Adverse events were collected and assessed according to the National Cancer Institute Common Terminology Criteria for Adverse Events (CTCAE) version 5.0.

### 2.4 Statistical analysis

Multivariate analysis was performed using the Cox proportional hazards model. Hazard ratios (HRs) and their 95% confidence intervals (CIs) were reported. Differences between groups were evaluated using the log-rank test. *P* value *< 0.05* was considered statistically significant. Categorical variables between the ICIs + Chemo group and the Chemo group were compared using Fisher’s exact test. PFS and OS were estimated using Kaplan–Meier survival curves. Data analysis and figure generation were performed using SPSS version 27.0.

The study was approved by the Ethics Review Committee of the People’s Hospital of Inner Mongolia Autonomous Region.

**Figure 1:**
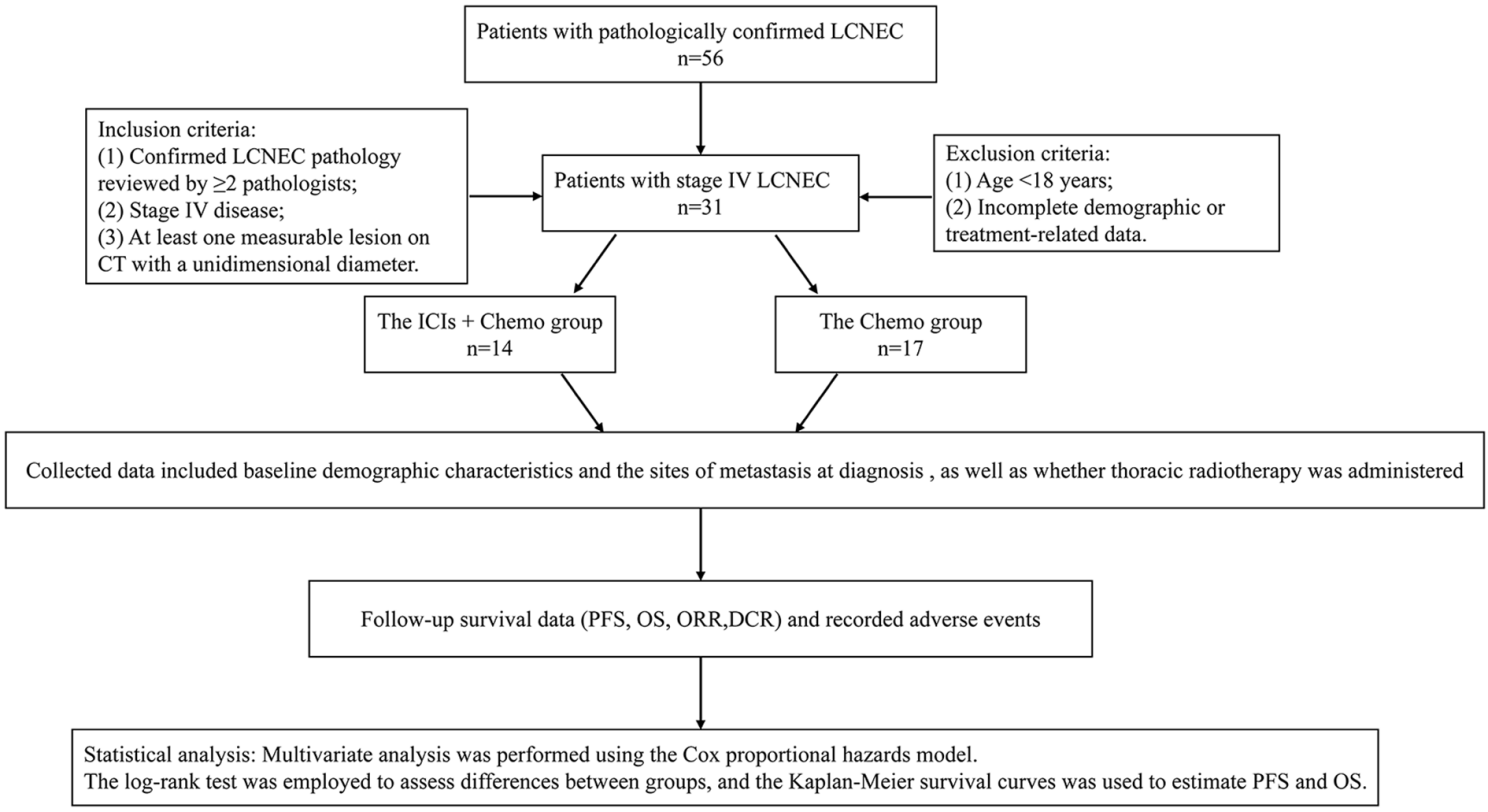
The enrollment criteria and analytical approach.

The patient flowchart is shown in **Figure 1**.

## 3 Results

### 3.1 Baseline characteristics of advanced LCNEC patients

With a median follow-up of 24 months, a total of 31 patients with advanced LCNEC were enrolled. Among them, 17 patients received etoposide plus platinum, irinotecan plus platinum, or nab-paclitaxel plus platinum as first-line chemotherapy (the Chemo group), while the remaining 14 patients were treated with ICIs combined with chemotherapy as first-line therapy (the ICIs + Chemo group). ICIs used included: PD-1 inhibitors (sintilimab, toripalimab, tislelizumab, serplulimab, penpulimab, and pembrolizumab); PD-L1 inhibitors (adebrelimab, atezolizumab, and durvalumab); and cadonilimab, a PD-1/CTLA-4 bispecific inhibitor.

The enrolled patients with advanced LCNEC were predominantly male smokers with an Eastern Cooperative Oncology Group (ECOG) performance status of 0–1 (**Table 1**). The ICIs + Chemo group included a higher proportion of patients aged over 65 years and with a history of smoking compared to the Chemo group. Furthermore, this group also had a higher prevalence of lung, liver, and brain metastases at diagnosis. No significant differences were observed in the baseline characteristics between the two treatment groups.

Detailed baseline characteristics are summarized in **Table 2**.

**Table 2:** Differences in clinical characteristics between the advanced LCNEC patients with chemo-immunotherapy and chemotherapy as first line.

Category and Sub-category	Total Patients (n=31)	Chemo-immune (n=14)	Chemotherapy (n=17)	*p* value
Age (year), mean ± SD	66.9 ± 8.45	70.4 ± 7.01	64.1 ± 8.63	0.034
Age ≥65, n (%)	22 (71.0)	12 (85.7)	10 (58.8)	0.131
Gender, Male, n (%)	28 (90.3)	12 (85.7)	16 (94.1)	0.560
ECOG PS, 0–1, n (%)	27 (87.1)	13 (92.9)	15 (88.2)	1.000
Smoking history, n (%)	26 (83.9)	12 (85.7)	14 (82.4)	1.000
Metastatic sites, n (%)				
- Bone	6 (19.4)	2 (14.3)	4 (23.5)	0.664
- Liver	7 (22.6)	3 (21.4)	4 (23.5)	1.000
- Brain	7 (22.6)	3 (21.4)	4 (23.5)	1.000
- Adrenal	2 (6.5)	1 (7.1)	1 (5.9)	1.000
- Pleura	8 (25.8)	4 (28.6)	4 (23.5)	1.000
- Intrapulmonary	7 (22.6)	5 (35.7)	2 (11.8)	0.192
-Other	3(9.7)	3 (21.4)	0	
Chemotherapy regimen, n (%)				
- EP/EC/EL	27 (87.1)	12 (85.7)	15 (88.2)	1.000
- IP	2 (6.45)	1 (7.1)	1 (5.9)	1.000
- TP	2 (6.45)	1 (7.1)	1 (5.9)	1.000
Thoracic radiotherapy, n (%)	3 (9.7)	1 (7.1)	2 (11.8)	1.000
Laboratory Parameters Comparison (Median [IQR])				
-LDH (IU/L)	278.00 (234.00–458.00)	278.00 (247.00–416.00)	279.00 (227.00–1511.00)	0.893
-Serum Sodium (mmol/L)	138.00 (136.30–139.40)	139.00 (137.00–139.40)	138.00 (135.00–140.00)	0.309
-CEA (ng/mL)	4.66 (2.17–11.45)	5.30 (2.54–11.45)	4.00 (1.42–55.80)	0.421
-pro-GRP (pg/mL)	53.52 (36.22–106.80)	53.52 (31.23–102.20)	54.40 (38.00–1551.00)	0.217
-NSE (ng/mL)	43.00 (27.45–59.36)	44.31 (20.47–59.36)	43.00 (32.00–109.00)	0.754

Values are mean ± SD, n (%). Range of normal values: Lactate dehydrogenase (LDH)(109–245 IU/L), Neuron specific enolase(NSE)(0.00-16.3 ng/mL), serum sodium (137–147 mmol/L), Pro-gastrin releasing peptide (pro-GRP): 0-77.8 pg/ml, Carcinoembryonic antigen (CEA):<5ng/ml. ECOG, Eastern Cooperative Oncology Group; IQR, intra quartile range; SD, standard deviation.

### 3.2 Treatment patterns in Advanced LCNEC patients

We compared the treatment responses between the two groups at the first efficacy evaluation. The results showed that among the 14 patients receiving first-line immuno-chemotherapy, ORR was 50% and DCR was 85.7%, which were higher than those in the Chemo group (29.4% and 76.5%, respectively); however, the differences were not statistically significant (*p > 0.05*).

Among patients with advanced LCNEC in the ICIs + Chemo group, five (35.71%) received PD-1 inhibitors, eight (57.14%) were treated with PD-L1 inhibitors, and one patient (7.14%) received a PD-1/CTLA-4 inhibitor.

Additionally, immune-related adverse events (irAEs) occurred in 28.56% of patients in the ICIs + Chemo group. All reported irAEs were grade 1–2 in severity, including two cases (14.28%) of immune-related hypothyroidism, one case (7.14%) of interstitial pneumonia, and one case (7.14%) of immune-related hepatitis. No grade 3 or higher adverse events were observed.

Detailed data are presented in **Table 3**.

**Table 3:** Treatment response and survival outcomes of advanced LCNEC patients in two groups.

Category and Sub-category	Chemo-immune (n=14)	Chemotherapy (n=17)	*p* value
Response at the first evaluation, n (%)			
- PR	7 (50.0)	5 (29.4)	
- SD	5 (35.7)	8 (47.1)	
- PD	2 (14.3)	4 (23.5)	
ORR(%)	50.0	29.4	0.285
DCR(%)	85.7	76.5	0.673
mPFS (months)	10.5	6.0	0.035*
1-Year PFS Rate (%)	42.9	5.9	
mOS (months)	15.0	11.0	0.036*
1-Year OS Rate (%)	54.5	50.0	
Ongoing Treatment, n	3	3	
ICIs, *n* (%)			
PD-1 inhibitors	5 (35.7)		
PD-L1 inhibitors	8 (57.1)		
PD-1/CTLA-4 bispecific	1 (7.1)		
irAEs (1-2), *n* (%)			
Interstitial lung disease	1 (7.1%)		
Hypothyroidism	2 (14.3%)		
Immune-mediated hepatitis	1 (7.1%)		

irAEs, immune-related inhibitors adverse events; ICIs, immune checkpoint inhibitors; DCR, disease control rate; ORR, objective response rate; PD, progressive disease; PR, partial response; SD, stable disease; mPFS, median Progression-free survival; mOS, median Overall survival. *p < 0.05.

### 3.3 Prognosis and survival analysis of Advanced LCNEC patients

**Figure 2:**
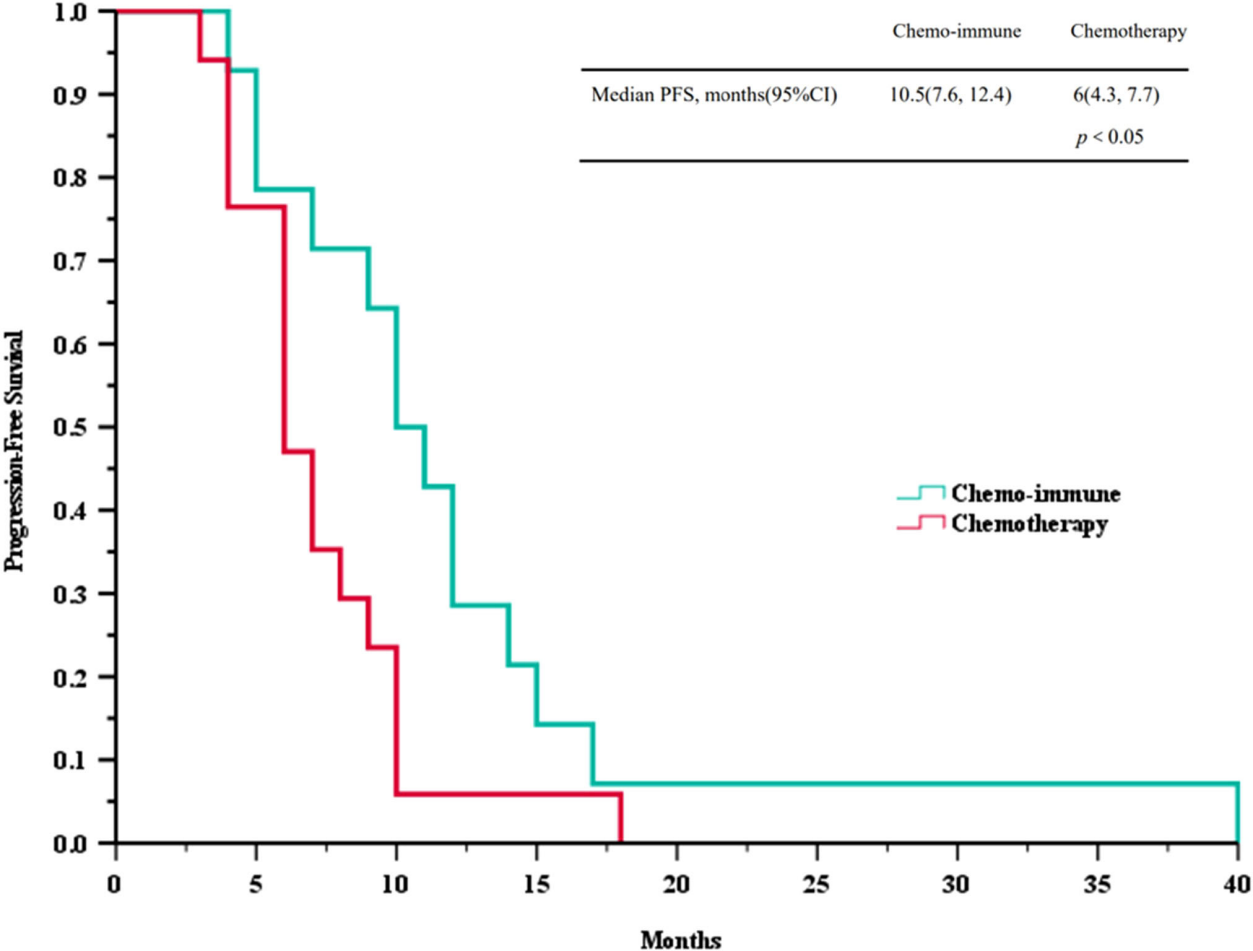
Survival curves of progression-free survival (PFS) between advanced LCNEC patients who chose immunotherapy combined with chemotherapy and chemotherapy as the first-line (FL) treatment.

**Figure 3:**
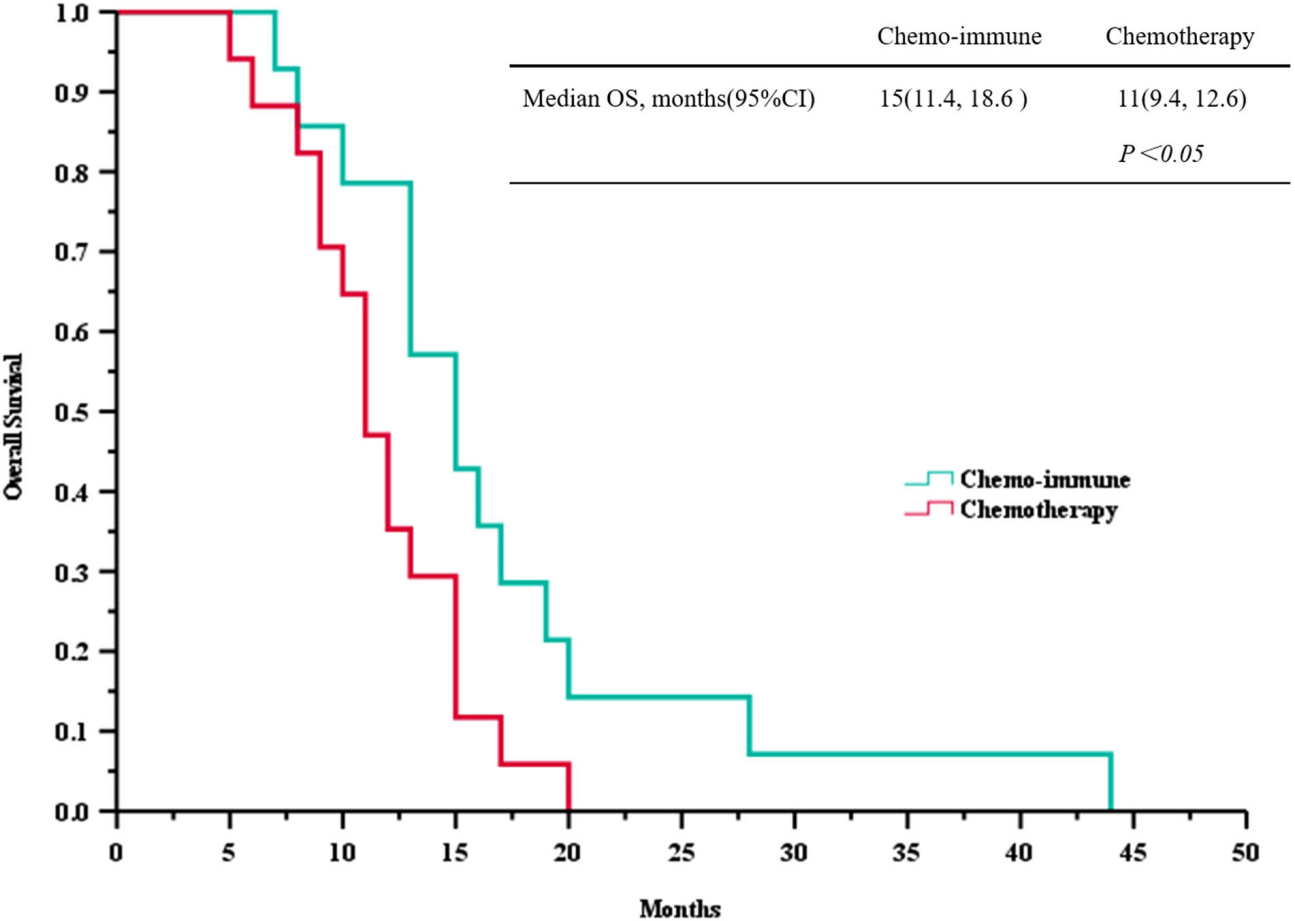
Survival curves of overall survival (OS) between advanced LCNEC patients who chose immunotherapy combined with chemotherapy and chemotherapy as the first-line (FL) treatment.

Based on the follow-up data, we plotted PFS and OS curves for both treatment groups (**Figure 2** and **Figure 3**). The median PFS in the ICIs + Chemo group was 10.5 months (95% CI, 7.6-12.4), significantly longer than that in the Chemo group(6.0 months; 95% CI, 4.3-7.7; *p* = 0.035). The median OS was also significantly prolonged in the ICIs + Chemo group (15.0 months; 95% CI, 11.4-18.6) compared with the Chemo group (11.0 months; 95% CI, 9.3-12.6; *p* = 0.036). The 1-year PFS rate was 42.9% and the 1-year OS rate was 78.8% in the ICIs + Chemo group, compared to 5.9% and 47.1%, respectively, in the Chemo group.

Multivariate analysis using the Cox proportional hazards model in 31 patients with advanced LCNEC showed that, compared to the Chemo group, the ICIs + Chemo group had a significantly decreased risk of disease progression by 49% (HR = 0.51; 95% CI, 0.28-0.92; *p=0.026*) and a reduced risk of overall mortality by 45% (HR = 0.55; 95% CI, 0.30-1.01; *p=0.054*). Only ECOG PS score (0–1 vs ≥2) was identified as an independent prognostic factor for PFS (HR = 1.48; 95% CI, 1.01-2.16; *p=0.043*), indicating that patients with better performance status had a significantly more favorable prognosis. Age, gender, smoking history, metastatic sites, and history of thoracic radiotherapy did not demonstrate significant effects (*p > 0.05*).

Detailed results are presented in **Table 4**.

**Table 4:** Multivariate analysis of advanced LCNEC patients on PFS and OS by Cox proportional hazard regression model.

Category and sub-category	PFS by multivariate analysis		OS by multivariate analysis	
	HR (95% CI)	*p* value	HR (95% CI)	*p* value
ICIs + chemo vs chemo	0.51 (0.28 – 0.92)	0.026*	0.55 (0.30 - 1.01)	0.054
Age, year ≥65vs.<65	1.02 (0.98 - 1.06)	0.280	1.01 (0.97 - 1.05)	0.685
Gender Male vs. Female	1.18 (0.54 - 2.60)	0.674	1.03 (0.46 - 2.30)	0.945
Smoking history Yes vs. No	1.32 (0.62 - 2.82)	0.475	1.06 (0.49 - 2.29)	0.880
PS scores 0–1 vs. ≥2	1.48 (1.01 - 2.16)	0.043*	1.41 (0.95 - 2.09)	0.086
Metastatic sites (Yes vs. No)				
- Bone	1.52 (0.73 - 3.16)	0.262	1.75 (0.83 - 3.71)	0.142
- Liver	1.55 (0.74 - 3.24)	0.246	1.38 (0.65 - 2.94)	0.398
- Brain	1.62 (0.77 - 3.41)	0.201	1.84 (0.86 - 3.91)	0.116
- Pleura	0.95 (0.47 - 1.92)	0.888	0.92 (0.45 - 1.88)	0.815
- Intrapulmonary	0.85 (0.42 - 1.71)	0.644	0.78 (0.38 - 1.60)	0.500
-Adrenal gland	1.20 (0.41 - 3.53)	0.740	1.05 (0.35 - 3.18)	0.931
Thoracic radiotherapy Yes vs. No	0.87 (0.37 - 2.04)	0.749	0.80 (0.34 - 1.89)	0.611

HR, hazard rate; PS, performance status; ICIs, immune checkpoint inhibitors. *p < 0.05.

## 4 Discussion

LCNEC is a rare and highly malignant neuroendocrine tumor, accounting for approximately 1–3% of all lung cancers. It is associated with a generally poor prognosis, with a 5-year survival rate of only about 15–30%. Treatment strategies for LCNEC are largely adapted from those for SCLC (17). Several phase III clinical trials have confirmed that ICIs combined with chemotherapy significantly improve PFS and OS in patients with ES-SCLC, providing significant survival benefit, ushering in a new era of immunotherapy for ES-SCLC, and have been recommended by Chinese CSCO guidelines as a first-line standard treatment for ES-SCLC (18).

Our findings are consistent with emerging evidence from other retrospective studies. For instance, Meng et al. (19) demonstrated that first-line chemotherapy combined with ICIs (atezolizumab or pembrolizumab) significantly improved the median OS of LCNEC patients compared with chemotherapy alone (56 weeks [95% CI, 22.2-89.8] vs. 28 weeks [95% CI, 16.3-39.7]; *p* = 0.029). Whereas the median PFS was 32 weeks (95% CI, 14.7-49.3) vs. 20 weeks (95% CI, 13.8-26.2), with no statistically significant difference (*p* = 0.136). Similarly, in a retrospective study by Song et al. (20) involving 10 patients with advanced LCNEC who received first-line pembrolizumab plus chemotherapy, the results showed an ORR of 70%, a DCR of 90%, a median PFS of 5.5 months (95%CI, 2.3-8.7), and a median OS of 13.0 months (95%CI, 11.0-15.0). In a larger analysis, Shirasawa et al. (21) analyzed 70 patients with advanced LCNEC and found that those who received anti-PD-1 therapy had significantly longer OS than those who did not (25.2 months[95% CI, 21.3-29.1] vs. 10.9 months[95% CI, 6.7-15.1], *p* = 0.02). Among the 13 patients treated with anti-PD-1 agents, 10 patients had negative PD-L1 expression, suggesting that ICIs may confer clinical benefit regardless of PD-L1 expression status. Collectively, these studies provide emerging clinical evidence supporting the efficacy of ICIs combined with chemotherapy in advanced LCNEC.

Due to the relative rarity of LCNEC in clinical practice, there is limited research on the application of ICIs in this malignancy. However, LCNEC is characterized by a high expression level of programmed death-ligand 1 (PD-L1) and a median tumor mutational burden (TMB) of 5.42 mutations per megabase (Mut/Mb), suggesting a biological rationale for the efficacy of immunotherapy in LCNEC (22–24). Epidemiological data indicated that the median OS for stage IV LCNEC patients is only 10 months, with a 5-year survival rate of less than 17% (25). A single-center retrospective analysis demonstrated that patients with advanced LCNEC treated with ICIs achieved a significantly longer median OS compared to those not receiving ICIs (23.5 months [95%CI, 18.524-28.476] vs. 11.2 months [95% CI, 4.530-18.930], *p* < 0.001) (26). For patients with advanced LCNEC, first-line treatment with ICIs combined with chemotherapy, following the therapeutic strategy for ES-SCLC, resulted in an overall ORR of 75% and a median PFS of 6.85 months. Comparisons with other retrospective analyses suggest that the treatment strategy for advanced LCNEC may be aligned with that for ES-SCLC (27). In summary, the current body of evidence suggests that ICIs combined with chemotherapy represent an effective first-line treatment strategy for patients with advanced LCNEC, potentially independent of PD-L1 expression status.

Our study contributes real-world data from Northern China on the management of advanced LCNEC. The analysis demonstrated that the ICIs + Chemo group achieved a median PFS of 10.5 months [95% CI, 7.6-12.4] (vs. 6 months [95% CI, 4.3-7.7] in the Chemo group) and a median OS of 15 months [95% CI, 11.4-18.6] (vs. 11 months [95% CI, 9.3-12.6] in the Chemo group). The combination of immunotherapy and chemotherapy significantly prolonged both PFS and OS in patients with advanced LCNEC (*p < 0.05*). IrAEs were generally mild (grade 1–2), with no occurrences of grade 3 or higher events. These results indicate that ICIs combined with chemotherapy may represent a promising first-line therapeutic option for patients with advanced LCNEC. Multivariate analysis using the Cox proportional hazards model demonstrated that treatment with ICIs combined with chemotherapy was an independent predictive factor associated with improved progression-free survival (PFS) (HR = 0.51; 95% CI, 0.28-0.92; *p=0.026*). For OS, a beneficial trend was also observed (HR = 0.55; 95% CI, 0.30-1.01; *p=0.054*). Additionally, better performance status (PS = 0-1) was identified as a favorable prognostic factor for prolonged PFS. No significant influences were detected for age, gender, smoking history, metastatic sites, or history of thoracic radiotherapy; further validation in larger cohorts is warranted. This study has several limitations, including its small sample size, single-center retrospective design, and potential selection bias. In addition, predictive biomarkers such as PD-L1 expression and tumor mutational burden were not incorporated into the analysis.

In summary, our study provides real-world clinical evidence indicating that ICIs combined with chemotherapy may represent a promising first-line therapy with the potential to provide survival benefit in patients with advanced LCNEC. Despite the limitations inherent to a single-center retrospective study and a small sample size, these preliminary findings offer valuable insight into the therapeutic potential of ICIs in advanced LCNEC and warrant further validation in multicenter, large-scale prospective clinical trials.

## Data Availability

The original contributions presented in the study are included in the article/**Supplementary Material**. Further inquiries can be directed to the corresponding author.
